# Validation of mandibular movements’ analysis to measure sleep in adults with sleep complaints by comparison with actigraphy and polysomnography

**DOI:** 10.5935/1984-0063.20220057

**Published:** 2022

**Authors:** Bassam Chakar, Frédéric Senny, Laurent Cambron, Anne-Lise Poirrier, Annick Bruwier, Farhad Baharloo, Robert Poirrier

**Affiliations:** 1 University Hospital of Liege, Sleep Study Center - Liège - Belgium; 2 Centre Hospitalier Régional de la Citadelle, Sleep Study Unit - Liège - Belgium; 3 High School HELMo-Gramme, Electronic and Informatic Unit - Liège - Belgium; 4 University Hospital of Liege, Department of Otorhinolaryngology - Liège - Belgium; 5 University Hospital of Liege, Department of Orthodontics and Dentofacial Orthopedics - Liège - Belgium

**Keywords:** Jaw, Actigraphy, Polysomnography, Mandible

## Abstract

**Objective:**

In adults with sleep complaints, we assessed the software of automatic analysis of mandibular movements to identify sleep and wake states by confrontation with the polysomnography (PSG) and the actigraphy (ACTG).

**Material and Methods:**

Simultaneous and synchronized in-lab PSG, ACTG, and JAWAC were carried out in 100 patients with a sleep complaint. Epoch by epoch analysis was realized to assess the ability to sleep-wake distinction. Sleep parameters as measured by the three devices were compared. These included three regularly reported parameters: total sleep time (TST), sleep onset latency (SOL), and wake after sleep onset (WASO). Also, two supplementary parameters, wake during sleep period (WDSP) and latency to arising (LTA) were added to measure separately the quiet wakefulness states.

**Results:**

The epoch by epoch analysis showed that the JAWAC, as compared to ACTG, classified sleep and wake states with greater specificity, while the overall accuracy and sensitivity of the two devices were comparable. The sleep parameters analysis showed that for the JAWAC estimates, the differences in TST, SOL, and LTA values were not statistically significant. However, WDSP and subsequently WASO were slightly underestimated. In contrast, the dissimilarities between ACTG estimates and PSG measurements of all the above sleep parameters were statistically significant; TST was overestimated whilst SOL, LTA, WDSP, and WASO were underestimated.

**Conclusion:**

This study indicated that, besides its ability to reliably estimate TST, the JAWAC based on mandibular movements’ analysis was able, in adults with sleep complaints, to overcome the important problem of the recognition of the state of quiet wakefulness.

## INTRODUCTION

Epidemiological studies carried out in the past decade have shown that obstructive sleep apnea (OSA) is a highly prevalent medical condition in the general population. It implies a range of clinical presentations with an ever-growing list of known adverse health consequences^1^. The diagnosis and severity of OSA are determined by the apnea-hypopnea index (AHI), which itself requires an accurate and objective assessment of both sleep time and respiratory events. Full in-lab polysomnography (PSG) is considered as the gold standard in the diagnosis of OSA, however this procedure can be costly and time-consuming limiting thus its scope. Consequently, alternative approaches based on portable devices used for home sleep testing (HST) were developed from early on^2^.

Whilst HST devices make use of different combinations of PSG respiratory sensors to identify respiratory events, they all lack the objective EEG measurement of the total sleep time (TST). Therefore, the International Classification of Sleep Disorders (ICSD) recommends that, in those conditions, the total recording time be used instead of the total sleep time in calculating respiratory disturbance index (RDI)^3^. This change of a variable, obviously, impacts both the diagnostic outcome and disease severity stratification^4^. To overcome this problem, many HST devices are using either a separate or an integrated actigraphy device (ACTG) as a surrogate for the EEG measurement of the TST. Although these devices improved the overall sensitivity and specificity of HST devices, they turn out to be less reliable in the presence of comorbidities, a situation frequently reported in patients who require a sleep study. The use of ACTG technology in association with HST devices is therefore considered as “conditional” rather than “standard” recommendation^5^,^6^. Otherwise, a growing number of HST devices resorts to technologies that do not make use of the same recommended variables for respiratory events as used in standard PSG, but instead rely on surrogate parameters. In such an approach, the number of channels used on a given device becomes less relevant than the sensitivity and specificity of the device and the clinical outcomes that it can achieve^7^.

One of the most promising surrogate used in HST is the analysis of the sagittal mandibular movement (MM) using a high-resolution magnetometer named JAWAC. Such analysis is able not only to recognize but also to differentiate between different sleep related respiratory events^8^. The algorithm used in the automatic analysis software of MM compared to PSG, proved to be a reliable alternative to the latter^9^. Moreover, as the sagittal mandibular movements reflected different behaviors associated, either to wakefulness (speaking, swallowing, eating, drinking, tonic support…), or to sleep (quiescence), we implemented another algorithm in order to detect sleep and wake epochs. This complementary algorithm provided a good estimation of the sleep and wake states^10^. The use of a single sensor to measure both the relevant respiratory and sleep parameters in a reliable way, offers a definite advantage that requires further validations. In a previous study conducted on healthy adults, we showed that MM was comparable to standard PSG and superior to ACTG in differentiating sleep and wake states^11^. However, the presence of sleep disturbances or comorbidities may interfere with the ability of a device to measure sleep. So, we designed the present study in order to assess the accuracy of predicting sleep and wake states by the analysis of MMs, confronted to synchronized analyses of ACTG and PSG, the latter one considered as the gold standard, in a cohort of patients suffering from a sleep disorder.

### Participants

The study was conducted at the Sleep Center of the University Hospital of Liege, Belgium. In accordance with the Helsinki declaration on human experimentation^12^, every participant read and signed an informed consent in which the aims of the study were also explained. They were selected from a group of adult patients who had been referred to the sleep center for investigation of sleep complaints. All patients were 18 years old or above. Their medical history, medications and demographic data were collected. The size of the cohort was limited to the first 100 patients in whom the simultaneous recordings of the devices were completed without technical defects.

### Measurements

#### Polysomnography

PSGs were carried out using EMBLA N7000 systems equipped with the Somnologica software. The PSG montage included three EEG channels, left and right EOG, chin EMG, bilateral tibialis anterior EMG, EKG, nasal cannula/pressure transducer, chest, and abdominal inductance plethysmography belts, fingertip pulse oximetry, snoring sensor, body position sensor, and light sensor. The manual scoring was done according to AASM scoring rules and was realized by qualified technologists blinded to the results of the other devices^2^. PSG was named hereafter as the gold standard for wake and sleep identification as well as for the diagnosis of sleep disorders.

#### Tested device: actigraphy (ACTG)

Actiwatch monitor (Actiwatch 2; Philips - Respironics, Murrysville, PA, USA) attached to the nondominant wrist was used for that purpose. Data were collected in 30-seconds epochs and analyzed thereafter by Philips ActiWare software version 6.0.1. The “default” settings provided by the manufacturer were selected for automatic analysis^13^.

#### Tested device: JAWAC

The JAWAC (Nomics - Liege, Belgium)^14^ is a device validated in the diagnosis of sleep breathing disorders through an analysis of mandibular movements. It employs a noninvasive motion sensor, based on the principle of electromagnetic self-induction. The output voltage at the receiver coil is a monotonic cubic function of the distance between the transmitter and the receiver coils. When the two coils are placed parallel to each other on the median-line of forehead and chin, the distance between them, which represents the sagittal MM, can be calculated from the properties of the received signal. The output was amplified, digitalized at a rate of 10Hz and made available online with the PSG channels. The data were also stored for subsequent retrieval and analysis. A first software based on MM analysis to detect and classify the ventilatory effort has been developed and validated^14^. Furthermore, a second validated software, using a wavelet-based complexity measure of the MM signal, was proposed to recognize sleep and wake states^10^.

#### Procedures

To ensure a reliable temporal synchronization between the three devices, we used the “Network Time Protocol”. Before each sleep study, the computer from each device was connected to the Internet and its clock synchronized manually with the Internet timeserver. Several units of each device were available for randomly use. Patients were admitted to the sleep laboratory between 14:00 and 17:00 hours. They were equipped early with ACTG and JAWAC sensors while those of PSG were installed later in the evening. Each patient freely chose the time devoted to sleep, in accordance with his or her bed and wake habits. The data from each device was stored for subsequent retrieval and analysis. At the end of each sleep study, the sleep technologist made sure that the three devices had remained synchronized, defined as showing no more than 30 seconds of discrepancy.

#### Data analysis

For methodological reasons, the duration of the recording was different with the three devices. Consequently, as they were all synchronized, we selected the period from “lights out” to “lights on” identified by the PSG sensors, as the time base for analysis for all three devices (gold standard PSG, tested device ACTG, and tested device JAWAC).

Qualified technologists scored the data from the PSG recordings manually and according to the AASM scoring rules. Automatic analyses were used in order to get ACTG and JAWAC data. The results of the scorings were available in 30sec. epochs. The PSG epochs were reduced to a binary form (S for any sleep stage and W for wakefulness), while those of ACTG and JAWAC were labeled directly ‘sleep (S)’ or ‘wake (W)’ by automatic analyses.

For each device, five derived sleep parameters were calculated using the same definitions. These included three AASM recommended parameters: 1) the total sleep time (TST) defined as the duration of all epochs labeled as sleep; 2) the sleep onset latency (SOL) measured as the time from light-off to the first epoch of sleep; and 3) the wake after sleep onset (WASO), which is the time scored as wake from first sleep epoch to light-on. Two additional parameters were added: 1) the wake during sleep period (WDSP), calculated as the time of wake between the first and the last epoch of sleep and 2) the latency to arising (LTA) measured as the elapsed time from last sleep epoch to light-on^15^.

#### Statistical analysis

We assessed the outcomes of the three devices both on a pooled-epoch and on a per-subject basis. The objectives of the statistical analysis were threefold: 1) to compare epoch by epoch the respective abilities of JAWAC and ACTG to differentiate sleep from wake states; 2) to compare their estimates of sleep parameters; and 3) to explore the differences between them.

In the epoch by epoch comparison, we combined all scored epochs from all subjects on a pooled-epoch basis and for each device. A three-way presentation of the results was used in accordance with the recommendations of the statistical guidance of the Food and Drug Administration (FDA)^16^.

The classification of the epochs by the PSG was used as reference. According to Tyron’s method^17^, sensitivity, specificity and accuracy were used to compute the percentage of matching epochs between each tested device and PSG. Sensitivity is the measure of the correctly identified sleep epochs. It is calculated by dividing the number of epochs correctly recognized by the tested device as sleep by the total number of PSG-identified sleep epochs. Specificity is the proportion of correctly identified wake epochs and is calculated by dividing the number of epochs the device correctly identified as wake by the total number of PSG-identified wake epochs. Accuracy is the overall agreement between PSG and the device. Accuracy is determined by dividing the cumulative number of correctly identified sleep and wake epochs by the total number of epochs in the recording period. Wake and sleep epoch agreements were analyzed for each device against PSG using the Cohen’s Kappa correlation, which determines the amount of agreement that can be expected by chance. This statistic ranges from 1, which demonstrates perfect agreement, to 0 which demonstrates agreement based on chance alone, and to -1 which demonstrates complete disagreement.

To investigate the differences between ACTG and JAWAC, we analyzed their agreements and disagreements. First, three agreement levels between ACTG and JAWAC were calculated: a) the overall agreement, is the percentage of the total number of epochs labeled identically by the two tested devices; b) the agreement in ‘sleep epochs’ is the percentage of identical labeling by the two tested devices in the epochs scored by the PSG as sleep; and c) the agreement in ‘wake epochs’ is the percentage of identical labeling by the two tested devices in the epochs scored by the PSG as wake. Second, in epochs where the two tested devices disagreed, we used the discrepant resolution test considering PSG as resolver to determine the ‘right’ device.

In the comparison between the estimates of sleep parameters, we proceeded to a per-subject analysis. The means and standard deviations of each sleep parameter were calculated for PSG, ACTG and JAWAC. The Pearson correlation coefficient, with 95% confidence interval was reported for the two devices against the PSG gold standard values. One-way ANOVA tests were used to verify if the ACTG and JAWAC estimates varied from the PSG measurement and Pairwise t-tests with adjusted *p*-values (using Holm-Bonferroni correction) were used to determine the significance of any difference between ACTG and JAWAC with the null hypothesis (the two devices provide equivalent performance). All statistically significant conclusions are made at an α=0.05 level.

To illustrate the data, we calculated for each subject the difference between the PSG measurements and the ACTG and JAWAC estimates. The mean differences (bias), the standard deviation of the differences and the limits of agreement were reported. A positive bias indicates an overestimation, and a negative bias indicates an underestimation relative to the PSG analysis, by the ACTG or JAWAC. For each sleep parameter, we displayed the Bland and Altman plots. Since PSG is the gold standard, we considered for each subject the PSG results rather than the mean of two methods to be plotted against the difference between PSG measurements and ACTG and JAWAC estimates^18^.

## RESULTS

One hundred seven sleep recordings were needed to obtain 100 simultaneous recordings unaffected by technical faults: 3 recordings were excluded for lack of synchronization, 1 for ACTG software problem, and 3 for JAWAC signal loss.

The demographic characteristics and sleep variables for the three devices are presented in Table 1. PSG was considered as normal in 6 patients (normal distribution and proportion of sleep stages; IAH <5; PLM <15/h). 42 patients were diagnosed with severe sleep apnea syndrome (IAH >30), 31 with moderate SAS (15<IAH<30) and 19 with mild SAS (5<IAH<15). Periodic limb movement (PLM >15/h) was present in 70 patients.

**Table 1:** Demographic and sleep parameters of the participants (n=100).

Variables	Value (mean ± SD)	Range
Gender (male/female)	59 : 41	
Age (years)	47.3 ± 14.4	19 - 87
Body mass index (kg/m^2^)	30.1 ± 5.9	17.5 - 49.1
Epworth sleepiness scale	11.7 ± 4.7	2 - 21
Hospital anxiety and depression scale (A-subscale)	9.1 ± 4.4	1 - 20
Hospital anxiety and depression scale (D-subscale)	7.1 ± 4	0 - 19
AHI (episodes/h)	31.3 ± 22.2	0.6 - 108.5
PLMI (episodes/h)	14.4 ± 9.8	1.1 - 57
Time in bed (min)	532.2 ± 91.7	210.5 - 771.5
Total sleep time (min), measured by PSG	405.6 ± 84.9	128.5 - 602.5
Total sleep time (min), estimated by ACTG	461.6 ± 95.2	135 - 648.5
Total sleep time (min), estimated by JAWAC	428.6 ± 97.9	4 - 687
Sleep efficiency (%), measured by PSG	60.3 ± 12.8	22.1 - 89.7
Sleep efficiency (%), estimated by ACTG	53.4 ± 12.2	0.5 - 84.5
Sleep efficiency (%), estimated by JAWAC	52.5 ± 13.1	18.9 - 85.4

### Performance of the sleep ⁄wake classification

In Table 2, a three-way presentation compares the sleep/wake classifications according to each device and the different combinations of labeling between ACTG and JAWAC for the overall epochs (106,456) and also for epochs scored by the PSG as sleep (81,169) or wake (25,287).

**Table 2:** A three-way presentation of sleep/wake classification comparing the ACTG, the JAWAC, and the PSG.

ACTG	JAWAC	Number of Epochs	PSG	
			Sleep	Wake
**Sleep**	**Sleep**	79,956	72,249	7,707
**Wake**	**Sleep**	5,784	2,249	3,535
**Sleep**	**Wake**	13,380	5,885	7,495
**Wake**	**Wake**	7,336	786	6,550
		106,456	81,169	25,287

The performance of ACTG and JAWAC compared to PSG are presented in Table 3. Both devices showed an identically high level of accuracy (82.86% for ACTG and 83.17% for JAWAC). The high sensitivity levels (96.26 for ACTG and 91.78 for the JAWAC) confirm their excellent ability to identify epochs of “sleep”. Whereas a higher specificity of JAWAC (55.54) compared with ACTG (39.88) indicates its greater efficiency in identifying epochs of “wake”. The JAWAC’s Cohen’s Kappa coefficient (0.50) even though moderate, was slightly higher than ACTG (0.43).

**Table 3:** Comparative performance on a pooled-epoch basis.

	ACTG		JAWAC	
	**Fractions**	**%**	**Fractions**	**%**
**Accuracy**	(72,249+5,885+3,535+6,550)/106,456	82.86	(72,249+2,249+7,495+6,550)/106,456	83.17
**Sensitivity**	(72,249+5,885)/81,169	96.26	(72,249+2,249)/81,169	91.78
**Specificity**	(3,535+6,550)/25,287	39.88	(7,495+6,550)/25,287	55.54
**Cohen’s Kappa**	0.43		0.50	

### Sleep parameters concordance

The sleep parameters calculated by each of the three devices are shown in Table 4. All ACTG estimates differed significantly from their corresponding PSG measures. TST was largely overestimated by ACTG, while the other parameters related to ‘wake’ were all underestimated.

**Table 4:** Sleep parameters on a per-subject basis as calculated by the three devices.

	PSG	ACTG		JAWAC	
	**Measures**	**Estimates**	**Correlation coefficient** **(95% confidence interval)**	**Estimates**	**Correlation coefficient** **(95% confidence interval)**
**TST**	405.6 ± 84.9	461.6 ± 95.2^ab^	0.64^*^ (0.54 , 0.88)	428.6 ± 97.9^b^	0.73^*^ (0.68 , 0.99)
**SOL**	32.4 ± 27.8	3.3 ± 3^ab^	0.11 (-0.01 , 0.33)	32.8 ± 51.8^b^	0.29^*^ (0.17 , 0.88)
**WASO**	94.3 ± 68.0	62.2 ± 35.7^a^	0.71^*^ (0.29 , 0.44)	70.9 ± 61.8^a^	0.54^*^ (0.34 , 0.64)
**WDSP**	83.2 ± 68.9	60.4 ± 35.1^a^	0.66^*^ (0.26 , 0.41)	61.9 ± 59.4^a^	0.54^*^ (0.32 , 0.61)
**LTA**	11.1 ± 16.7	1.8 ± 2.2^ab^	0.13 (-0.09 , 0.04)	9 ± 12.1^b^	0.69^*^ (0.39 , 0.60)

Notes: Statistically significant inter-instrument results are marked in the table: *p<0.05; ^a^Significant difference from PSG measures; ^b^Significant difference between ACTG and JAWAC estimates.

The Pearson correlation coefficient between ACTG and PSG showed a good and significant correlation for TST, WASO, and WDSP and poor or non-significant correlation for SOL and LTA.

The JAWAC’s estimates of TST and SOL were not significantly different from the PSG measurements, while WASO/wake time was underestimated and significantly different. Interestingly, a differentiation of WASO into its two components, WDSP and LTA, showed that this difference is due to the WDSP, which is underestimated rather than to the LTA, which does not show a significant difference. The correlation coefficient for the JAWAC estimates showed a significant correlation for all the parameters with a good correlation for TST and LTA and a modest one for SOL, WASO, and WDSP.

### Bias and precision statistics

**Figure 1:**
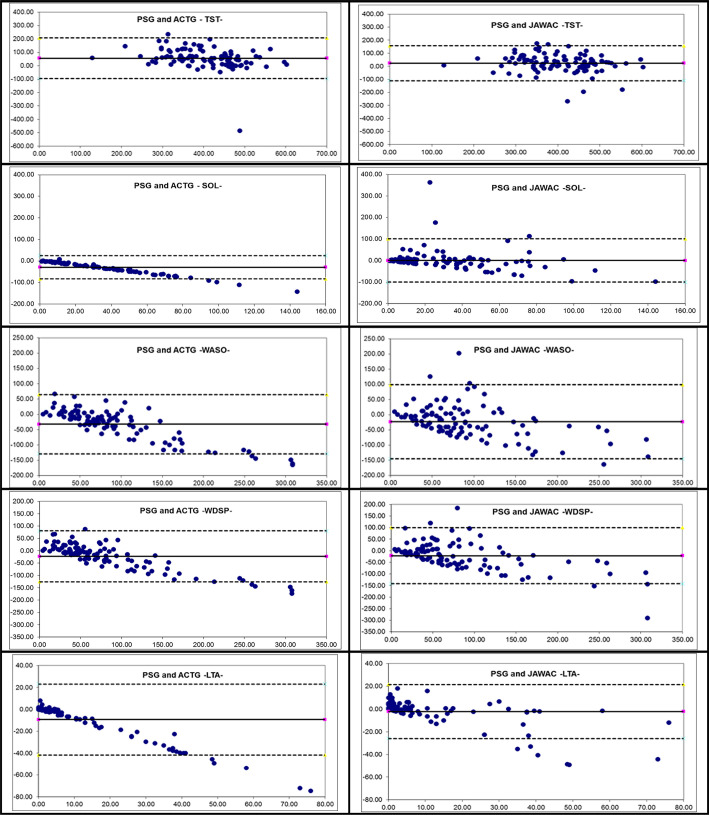
Bland-Altman plots for TST (total sleep time), SOL (sleep onset latency), WASO (wake after sleep onset), WDSP (wake during sleep period), and LTA (latency time to arising).

Notes: ( - ) Mean difference; X - axis reflects the mean of the PSG and the device.

The Bland and Altman statistics for the sleep parameters are described in Table 5 and the plots are shown in Figure 1. According to these results, the directions of the biases were the same for both devices: an overestimation of TST by the 2 tested devices but the overestimation with the JAWAC was less than half the one for the ACTG. Moreover, the JAWAC expressed a rather adequate estimation of SOL. The other wake parameters were underestimated by the two tested devices but far less for the LTA, with the JAWAC. The biases with JAWAC were closer to zero and with more tightened limits of agreement for all sleep parameters and showed a greater degree of constancy with regard to TST, SOL, and LTA. ACTG on the other hand showed a constant bias only with regard to TST whereas they tended to diverge farther as the values for PSG increased.

( ---- ) 2 Standard deviations; Y - axis is denote the difference between PSG and the device.

**Table 5:** Bland and Altman plot statistics.

	ACTG		JAWAC	
	**Mean ± SD**	**ULOA; LLOA**	**Mean ± SD**	**ULOA; LLOA**
**TST**	56.0 ± 77.4	207.6 ; -95.7	23.0 ± 68.3	156.9 ; -111.0
**SOL**	-29.2 ± 27.6	25.0 ; -83.3	0.4 ± 51.4	101.1 ; -100.2
**WASO**	-32.0 ± 49.4	64.8 ; -128.9	-23.4 ± 62.3	98.6 ; -145.4
**WDSP**	-22.8 ± 52.7	80.5 ; -126.1	-21.3 ± 61.9	100.1 ; -142.7
**LTA**	-9.3 ± 16.5	23.1 ; -41.6	-2.1 ± 12.1	21.6 ; -25.8

Notes: Analyses were conducted on the difference between PSG measurements and ACTG and JAWAC estimates. Negative value of mean indicate underestimation; positive value of mean indicate overestimation; SD = Standard deviation; ULOA; LLOA = Upper and lower limits of agreement.

### Exploring the differences between ACTG and JAWAC

Table 6 presents on a pooled-epoch basis, the analysis of agreements and disagreements between ACTG and JAWAC in classifying epochs into sleep and wake. These results showed a good level of agreement (0.82) between the two devices, and where they agreed, their degree of convergence with the reference PSG scoring was excellent (0.90). We used the discrepant analysis of disagreement with PSG as a resolver to analyze the disagreements between ACTG and JAWAC^16^. This showed that both devices overall produced the same level of agreement with PSG (0.49 for ACTG and 0.51 for JAWAC). ACTG was more accurate in correctly identifying the sleep epochs (0.72). Conversely, JAWAC was more accurate in correctly identifying wake epochs (0.68).

**Table 6:** Analysis of agreement and disagreement between ACTG and JAWAC in a pooled-epoch basis.

	Fractions	Percentages
	**Agreement analysis**	
**Overall agreement**	(79,956+7,336)/106,456	82
ACTG & JAWAC agree and are both correct	(72,249+6,550)/(79,956+7,336)	90
ACTG & JAWAC agree and are both wrong	(7,707+786)/( 79,956+7,336)	10
**Overall disagreement**	(5,784+13,380)/106,456	18
	**Discrepant analysis of disagreement - PSG as resolver**	
**Overall epochs with disagreement**		
ACTG agree with PSG	(5,885+3,535)/( 5,784+13,380)	49
JAWAC agree with PSG	(2,249+7,495)/(5,784+13,380)	51
**Sleep’s epochs**		
ACTG agree with PSG	5,885/(2,249+5,885)	72
JAWAC agree with PSG	2,249/(2,249+5,885)	28
**Wake’s epochs**		
ACTG agree with PSG	3,535/(3,535+7,495)	32
JAWAC agree with PSG	7,495/(3,535+7,495)	68

The sleep parameters (Table 4) demonstrate the presence of a significant difference between ACTG and JAWAC estimates of TST but not those of WASO, although when this is broken down into its two components, LTA and WDSP, a significant difference is found with regard to the former but not to the latter.

## DISCUSSION

In the current study, we sought to assess the performance of the JAWAC, a new HST device based on MM analysis, to identify wake and sleep state and provide estimates of sleep parameters.

We chose to include patients who were referred to the sleep laboratory for a PSG, regardless of the nature of the suspected sleep disorder, so as to minimize the impact of a given sleep disorder on the performance of the device^6^.

We also compared the results of JAWAC with those of both the reference standard (PSG) and the non-reference standard (ACTG). This triangular comparison allowed a more accurate assessment of the differences between JAWAC and ACTG.

Our results showed that JAWAC, as compared to ACTG, classified sleep and wake states with greater specificity, while the overall accuracy and sensitivity of the two devices were comparable. Furthermore, the use of PSG as the determining factor in disagreements between the two HST devices showed a superiority of JAWAC in correctly identifying the wake epochs. The fact that wake occurs more frequently in patients with sleep breathing disorders as compared to normal subjects, regardless of its distribution within the TIB, may explain the greater impact of the degree of specificity of such surrogate devices, as we have defined above, on the quality of the sleep analysis in those patients.

In the choice of parameters, we used TST as a specific measure of the duration of sleep. We also made a distinction between different situations of wakefulness during TIB, by designating SOL and LTA as two periods of quiet wake, distinct from WDSP, which is a measure of the time awake during the sleep period. WASO was calculated by adding WDSP to LTA. The sleep efficiency, which is the ratio of TST to TIB, was reported in table 1 but not included in the statistical analysis due to the fact that the denominator was common for the three devices.

Set against PSG as reference, JAWAC proved efficient in distinguishing sleep from quiet wakefulness. This was illustrated by the fact that the differences in TST, SOL, and LTA values were not statistically significant. However, WDSP and subsequently WASO were slightly underestimated.

In contrast, the dissimilarities between ACTG estimates and PSG measurements of all the above sleep parameters were statistically significant; TST was overestimated whilst SOL, LTA, WDSP, and WASO were underestimated. These results of the ACTG performance are broadly in agreement with earlier reports, which show that actigraphies, regardless of the manufacturer and software, tend to rate quiet wakefulness as sleep, and hence systematically overestimate TST and underestimate SOL and WASO^5^.

The results presented in this study indicated that, besides its ability to reliably estimate TST, JAWAC was able to overcome the important problem of the recognition of the state of quiet wakefulness.

The better performance of JAWAC in this regard is probably related to the distinct behavior of mandibular muscles during sleep. The position of the mandible is the result of a balance of forces between the jaw-closing and the jaw-opening muscles. The onset of sleep has different effects on the basal activity of those muscles, where masseter and medial pterygoid show a significant decrease in their tonic activities whereas genioglossus and geniohyoid muscles maintain a greater phasic activity^19^. The resultant imbalance induces thus an opening movement of the jaw that is recorded both in healthy adults and patients with obstructive sleep apneas syndrome^20^,^21^.

The automatic analysis algorithm used in JAWAC recognizes the onset of sleep from a decrease in the amplitude of the jaw movements associated with a slight mouth opening lasting for at least 2 minutes, whereas the onset of wakefulness is recognized from a sharp increase in the amplitude of movements^10^. The ability of JAWAC to efficiently detect these specific mandibular movements probably explains its success in identifying the state of quiet wakefulness.

This study has however some limitations, which ought to be taken into account in future research. Just as it has been shown in the literature that actigraphies’ ability to measure sleep may be affected to different extent within different sleep disorders. Further studies should be carried out to evaluate the performance of JAWAC in specific patient groups, such as in those suffering from insomnia and sleep breathing disorders. Furthermore, in the choice of sleep parameters it seems appropriate to take into account sleep efficiency, as this parameter is widely used in validation studies.

## CONCLUSION

Our study demonstrates the ability of JAWAC to correctly identify sleep/wake epochs and thus give an accurate estimation of various sleep parameters. This feature combined with its capacity to record sleep respiratory events, validated in previous studies, makes it a device unique in its ability to calculate AHI reliably using a single sensor, a valuable asset in the science of HSTs that seeks to couple simplicity with reliability.

## References

[1] McNicholas WT, Bonsignore MR, Lévy P, Silke R (2016). Mild obstructive sleep apnoea: clinical relevance and approaches
to management. Lancet Respir Med.

[2] Kapur VK, Auckley DH, Chowdhuri S, Kuhlmann DC, Mehra R, Ramar K (2017). Clinical practice guideline for diagnostic testing for adult
obstructive sleep apnea: an American Academy of Sleep Medicine Clinical
Practice Guideline. J Clin Sleep Med.

[3] American Academy of Sleep Medicine (AASM) (2014). International classification of sleep disorders.

[4] Escourrou P, Grote L, Penzel T, McNicholas WT, Verbraecken J, Tkacova R (2015). The diagnostic method has a strong influence on classification of
obstructive sleep apnea. J Sleep Res.

[5] Van de Water ATM, Holmes A, Hurley DA (2011). Objective measurements of sleep for non-laboratory settings as
alternatives to polysomnography - a systematic review. J Sleep Res.

[6] Smith MT, McCrae CS, Cheung J, Martin JL, Harrod CG, Heald JL (2018). Use of actigraphy for the evaluation of sleep disorders and
circadian rhythm sleep-wake disorders: an American Academy of Sleep Medicine
clinical practice guideline. J Clin Sleep Med.

[7] Sands SA, Owens RL, Malhotra A (2016). New approaches to diagnosing sleep-disordered
breathing. Sleep Med Clin.

[8] Martinot JB, Le-Dong NN, Cuthbert V, Denison S, Silkoff PE, Guénard H (2017). Mandibular movements as accurate reporters of respiratory effort
during sleep: validation against diaphragmatic
electromyography. Front Neurol.

[9] Senny F, Destiné J, Poirrier R (2008). Midsagittal jaw movements analysis for the scoring of sleep
apneas and hypopneas. IEEE Trans Biomed Eng.

[10] Senny F, Maury G, Cambron L, Leroux A, Destiné J, Poirrier R (2012). The sleep/wake state scoring from mandible movement
signal. Sleep Breath.

[11] Chakar B, Senny F, Poirrier AL, Cambron L, Fanielle J, Poirrier R (2017). Validation of midsagittal jaw movements to measure sleep in
healthy adults by comparison with actigraphy and
polysomnography. Sleep Sci.

[12] World Medical Association (WMA) (2013). World Medical Association Declaration of Helsinki: ethical
principles for medical research involving human subjects. JAMA.

[13] O’Hare E, Flanagan D, Penzel T, Garcia C, Frohberg D, Heneghan C. A (2015). comparison of radio-frequency biomotion sensors and actigraphy
versus polysomnography for the assessment of sleep in normal
subjects. Sleep Breath.

[14] Beckers B, Poirrier R, Destine J (2000). In: Proceeding 1st Annual International IEEE-EMBS - Special Topic
Conference Microtechnologies in Medicine and Biology.

[15] Kushida CA, Littner MR, Morgenthaler T, Alessi CA, Bailey D, Coleman J (2005). Practice parameters for the indications for polysomnography and
related procedures: an update for 2005. Sleep.

[16] Food and Drug Administration (FDA) Statistical guidance on reporting results from studies evaluating
diagnostic tests [Internet].

[17] Tyron WW, Bellack AS, Hersen M (1991). Activity measurement in psychology and medicine.

[18] Krouwer JS (2008). Why Bland-Altman plots should use X, not (Y+X)/2 when X is a
reference method. Stat Med.

[19] Fogel RB, Trinder J, White DP, Malhotra A, Raneri J, Schory K (2005). The effect of sleep onset on upper airway muscle activity in
patients with sleep apnoea versus controls. J Physiol.

[20] Miyamoto K, Ozbek MM, Lowe AA, Sjöholm TT, Love LL, Fleetham JA (1998). Mandibular posture during sleep in healthy adults. Arch Oral Biol.

[21] Miyamoto K, Ozbek MM, Lowe AA, Sjöholm TT, Love LL, Fleetham JA (1999). Mandibular posture during sleep in patients with obstructive
sleep apnoea. Arch Oral Biol.

